# Differential Subcellular Localization Renders HAI-2 a Matriptase Inhibitor in Breast Cancer Cells but Not in Mammary Epithelial Cells

**DOI:** 10.1371/journal.pone.0120489

**Published:** 2015-03-18

**Authors:** Hsiang-Hua D. Chang, Yuan Xu, Hongyu Lai, Xiaoyu Yang, Chun-Che Tseng, Ying-Jung J. Lai, Yu Pan, Emily Zhou, Michael D. Johnson, Jehng-Kang Wang, Chen-Yong Lin

**Affiliations:** 1 Lombardi Comprehensive Cancer Center, Department of Oncology Georgetown University Washington, DC, 20057, United States of America; 2 Department of Biochemistry, National Defense Medical Center, Taipei, Taiwan, ROC; 3 Department of Biology, Carleton College, Northfield, MN, 55057, United States of America; 4 Thomas Jefferson High School for Science and Technology, Alexandria, VA, 22046, United States of America; Hormel Institute, University of Minnesota, UNITED STATES

## Abstract

The type 2 transmembrane serine protease matriptase is under tight control primarily by the actions of the integral membrane Kunitz-type serine protease inhibitor HAI-1. Growing evidence indicates that HAI-2 might also be involved in matriptase inhibition in some contexts. Here we showed that matriptase inhibition by HAI-2 depends on the subcellular localizations of HAI-2, and is observed in breast cancer cells but not in mammary epithelial cells. HAI-2 is co-expressed with matriptase in 21 out of 26 human epithelial and carcinoma cells examined. HAI-2 is also a potent matriptase inhibitor in solution, but in spite of this, HAI-2 inhibition of matriptase is not observed in all contexts where HAI-2 is expressed, unlike what is seen for HAI-1. Induction of matriptase zymogen activation in mammary epithelial cells results in the formation of matriptase-HAI-1 complexes, but matriptase-HAI-2 complexes are not observed. In breast cancer cells, however, in addition to the appearance of matriptase-HAI-1 complex, three different matriptase-HAI-2 complexes, are formed following the induction of matriptase activation. Immunofluorescent staining reveals that activated matriptase is focused at the cell-cell junctions upon the induction of matriptase zymogen activation in both mammary epithelial cells and breast cancer cells. HAI-2, in contrast, remains localized in vesicle/granule-like structures during matriptase zymogen activation in human mammary epithelial cells. In breast cancer cells, however, a proportion of the HAI-2 reaches the cell surface where it can gain access to and inhibit active matriptase. Collectively, these data suggest that matriptase inhibition by HAI-2 requires the translocation of HAI-2 to the cell surface, a process which is observed in some breast cancer cells but not in mammary epithelial cells.

## Introduction

Interactions between a protease and a protease inhibitor that can be observed in solution may be irrelevant in whole cells and particularly *in vivo*. The significant structural homologies among the catalytic domains of proteases and among the reactive center of protease inhibitors mean that many inhibitors can bind to and inhibit proteases that they would never normally encounter in nature. The identification of artifactual protease-inhibitor complexes can be a pitfall of common experimental strategies; for example protease inhibitors identified using purified active protease in solution. One example of this involves the protein products of the *SPINT1* and *SPINT2* genes, which encode two highly related, integral membrane, Kunitz-type serine protease inhibitors, named hepatocyte growth factor (HGF) activator inhibitor type (HAI)-1 and 2 [1,2]. As indicated by their nomenclature, HAI-1 and HAI-2 which are expressed predominantly by epithelial cells [3,4], have been shown to act against HGF activator (HGFA), a predominantly liver-derived, blood-borne serine protease [5]. While the role of HAI-1 in the control of HGFA *in vivo* remains the subject of debate due to the expression of these proteins by different cell types with different subcellular localization, considerable evidence does indicate that the type 2 transmembrane serine protease matriptase is the genuine physiological target protease of HAI-1. Stable matriptase-HAI-1 complexes were initially isolated from human milk [6] and have been detected in other body fluids [7]. In addition to being a potent matriptase inhibitor with a Ki of the order of nM [8] and the widespread co-expression of the inhibitor with matriptase in epithelial tissues [3,4,9], HAI-1 also plays an important role in matriptase synthesis, intracellular trafficking and zymogen activation [10,11].

HAI-2 resembles HAI-1 in many regards, suggesting that HAI-2 may also be a physiological matriptase inhibitor [4]. In addition to the similarity of their protein domain structures with a transmembrane domain and two Kunitz domains, the amino acid sequence flanking the reactive site loop of the Kunitz domain 1 in HAI-2 is almost identical to that in HAI-1, suggesting that HAI-2 can inhibit proteases with similar inhibitory specificity to HAI-1. Indeed, soluble recombinant human HAI-2 exhibits similar inhibition potency to that of soluble recombinant human HAI-1 against recombinant matriptase serine protease domain, and both inhibitors form stable complexes with matriptase [4]. HAI-2 is also broadly expressed by epithelial cells, in which HAI-1 and matriptase are also expressed [4]. The hypothesis that HAI-2 is a physiological inhibitor of matriptase has been further bolstered by the observation that matriptase ablation can reverse the defects in placenta development caused by the targeted deletion of either HAI-1 or HAI-2 in the mouse [12].

Although HAI-2 may be a genuine physiological inhibitor of matriptase in the mouse, the relationship between matriptase and HAI-2 in human is much less clear than that between matriptase and HAI-1. Induction of matriptase zymogen activation in epithelial and carcinoma cells results in the formation of matriptase-HAI-1 complexes [13]. It is less certain that matriptase-HAI-2 complexes are also formed during this process. Furthermore, the data from mouse models for a functional relationship between matriptase and HAI-2 may not be relevant for human matriptase and HAI-2, suggesting that there could be genuine physiological difference in the role of HAI-2 in human *versus* rodents. Targeted deletion of HAI-2 in mice results in impaired placental development, leading to embryonic lethality [12]. In contrast, no obvious embryonic or developmental defects appear to be associated with the loss of HAI-2 in patients with the syndromic form of congenital sodium diarrhea [14]. This physiological difference may, in part, be attributed to the fact that the predominant HAI-2 species expressed by mouse epithelial tissues lacks the first Kunitz domain that is responsible for matriptase inhibition [15]. As a consequence, mouse HAI-2 might not effectively inhibit some serine proteases, including HGFA and matriptase, as does human HAI-2. In the current study, we evaluated the functional link between human matriptase and both human HAI-1 and HAI-2 by analyses of the expression of these proteins by various epithelial cells, their biochemical interactions in solution and in intact cells, and their subcellular distribution during the course of matriptase zymogen activation. Our study indicates that while HAI-2 possesses biochemical capacity to be an effective matriptase inhibitor, the subcellular localization of these proteins determines whether HAI-2 has access to active matriptase.

## Materials and Methods

### Chemicals and reagents

5,5’-Dithio-bis-(2-Nitrobenzoic Acid) (DTNB) and 4',6-diamidino-2-phenylindole (DAPI) were obtained from Sigma-Aldrich (St. Louis, MO); N-tert-butoxycarbonyl (Boc)-Gln-Ala-Arg-7-Amido-4- methylcoumarin (AMC) was purchased from Enzo Life Sciences (Farmingdale, NY); Fetal bovine serum (FBS) was obtained from Omega Scientific (Tarzana, CA).

### Cell cultures

184 A1N4 (a gift from M. R. Stampfer, UC Berkeley) [16] human mammary epithelial cells were cultured in a modified Improved Minimum Essential Medium (IMEM) supplemented with 0.5% FBS, 5 μg/ml recombinant human insulin (rh-insulin) (Invitrogen), 5 μg/ml hydrocortisone (Sigma), and 10 ng/ml recombinant human epidermal growth factor (rhEGF) (Promega). MTSV-1.1 B and MTSV-1.7 milk-derived human mammary epithelial cells (a gift from Dr. J. Taylor-Papadimitriou (Imperial Cancer Research Fund, London) [17], MCF-7 (ATCC), T-47D (ATCC), MDA-MB-231 (ATCC), SK-BR-3 (ATCC), and BT-549 (ATCC) human breast cancer cells, Colo 357 (ATCC) pancreatic adenocarcinoma cells, and JEG-3 (ATCC) human placental choriocarcinoma cells were cultured in IMEM supplemented with 10% FBS. RWPE-1 (ATCC) human prostate epithelial cells were cultured in keratinocyte serum free medium (Invitrogen) supplemented with 50 μg/ml bovine pituitary extract and 5 ng/ml rhEGF. LNCaP (ATCC), CWR22 (ATCC), DU145 (ATCC), and PC3 (ATCC) prostate cancer cells were cultured in RPMI-1640 medium supplemented with 10% FBS. HaCaT (CLS Cell Lines Service GmbH, Eppelheim Germany) human keratinocytes, SCC-25/CP (University of Pittsburgh) [18], PCI-51 (University of Pittsburgh) [19,20], JHU-011 and JHU-028 (Johns Hopkins University) human head and neck squamous carcinoma cells, A549 (ATCC) human lung carcinoma cells, Hep G2 (ATCC) human hepatocellular carcinoma cells and human colon carcinoma cells, Caco2 (ATCC), HT-29 (ATCC), Lovo (ATCC), and LS 174T (ATCC) were cultured in DMEM supplemented with 10% FBS. The cells were incubated at 37°C in a humidified atmosphere with 5% CO_2_.

### Monoclonal antibodies

The HAI-2 mAb DC16 was generated by conventional immunization and hybridoma fusion methods using soluble recombinant HAI-2 (Novoprotein, Summit NJ) as the immunogen. The mouse monoclonal antibodies M24 and M19 were used for immunoblot analyses to detect total matriptase and HAI-1, respectively [13,21,22]. The matriptase mAb 21-9, HAI-1 mAb M19, and HAI-2 mAb DC16 immobilized on Sepharose beads were used for the immunodepletion.

### Acid-induced matriptase zymogen activation

For the adherent cells, the cells were washed with PBS three times and then incubated with 150 mM phosphate buffer pH 6.0 at room temperature for 20 min as previously describe [21]. The cells were then washed with PBS once and lysed in 1% Triton X-100 and 1 mM DTNB in PBS for immunoblot analysis. For Ramos human Burkitt lymphoma cells which grown in suspension, the cells were washed with PBS and then incubated with 150 mM phosphate buffer pH 6.0 at a cell density of about 1.4x 10^6^ cells/ml at room temperature for 20 min. The cells were then spun down by centrifugation and the supernatant was collected as the shed fraction, which contains active matriptase.

### Inhibition of and complex formation with active matriptase by HAIs in solution

HAI-1 and HAI-2 were prepared from 184 A1N4 cells. The cells were lyzed in 1% Triton and 1 mM DTNB in PBS. The cell lysates were mixed with matriptase mAb 21-9-Sepharose and incubated at 4°C for 2 hours to deplete matriptase. The matriptase-depleted cell lysate was mixed with the shed fraction collected from acid-activated Ramos cells. The mixture was then incubated in a water bath at 37°C for 10 min and then assayed for tryptic activity assay, and subjected to immunodepletion, and immunoblot analysis.

### Tryptic activity assay

The matriptase activity was assessed by measuring 7-Amino-4-methylcoumarin (AMC) released from a synthetic fluorogenic substrate Boc-Gln-Ala-Arg-AMC in microfluor 96-well black microtiter plates (Thermo Scientific). The reaction buffer contained the synthetic substrate with a final concentration of 125 μM, 100 mM Tris buffer pH 8.5, and samples and distilled water added to a total volume of 200 μl. The cleavage of the synthetic fluorogenic substrate was recorded using a Wallac 1420 Victor 2 microplate reader with an excitation wavelength of 360 nm and detecting emission at 480 nm.

### Western blotting

Cells were lysed in PBS containing 1% Triton X-100 and 1 mM DTNB. DTNB was added to the lysis buffer to prevent the cleavage of disulfide linkages [23]. The protein concentration of the lysates was determined by Bradford protein assay and equal amounts of proteins or equal proportions of cell lysates and conditioned buffers were analyzed by Western blot. Protein samples were diluted in 5x SDS sample buffer containing no reducing agent and incubated at room temperature for 5 min prior to loading onto the gels. Proteins were resolved by 7.5% SDS-PAGE, transferred to nitrocellulose membranes, and probed with the indicated mAbs. The binding of mAbs was detected using HRP conjugated secondary antibodies, and visualized using Western Lightening Chemiluminescence Reagent Plus (Perkin-Elmer, Boston, MA).

### Immunodepletion

The matriptase mAb 21–9, HAI-1 mAb M19, and HAI-2 mAb DC16 were covalently coupled to Sepharose 4B at 5 mg/ml gel as described previously [13]. For immunodepletion, samples (200 μl) were incubated with 15 μl of the mAb conjugated Sepharose and rotated in cold room for 2 hours. Supernatants were separated from the beads by centrifugation and collected as the immunodepleted fraction.

### Immunofluorescence

Cells were cultured on 18 mm circular cover slides in 12-well plate. For induction of matriptase zymogen activation, 184 A1N4 mammary epithelial cells were stimulated with fresh culture medium for 30 min [24,25] and the breast cancer cells were treated with 150 mM phosphate buffer pH 6.0 for 20 min. The cells were fixed in 10% buffered formalin (Fisher Scientific) for 20 min. The cells were either not permeabilized or permeabilized with 0.5% Triton in PBS buffer for 5 min. For indirect immunofluorescent staining, the cells on cover slides were incubated with the primary mAbs at 2 μg/ml at room temperature for 20 min followed by Alexa Fluor 555 goat anti-mouse IgG for 30 min. Alexa Fluor 488 nm conjugated phalloidin for F-actin staining and DAPI for the nucleus were used as counterstains. For double labeling studies, the cells were firstly stained with unlabeled primary mAbs followed by Alexa Fluor 555 goat anti-mouse IgG, and then 5–10 μg/ml Alexa Fluor 488 nm conjugated HAI-2 mAb DC16 in a solution with 0.5 mg/ml mouse IgG for 30 min. Images were captured using a Zeiss LSM 510 confocal microscope.

## Results

### Expression of matriptase is tightly correlated with HAI-1 expression, and to a lesser extent with HAI-2, in human epithelial and carcinoma cells

**Fig 1:**
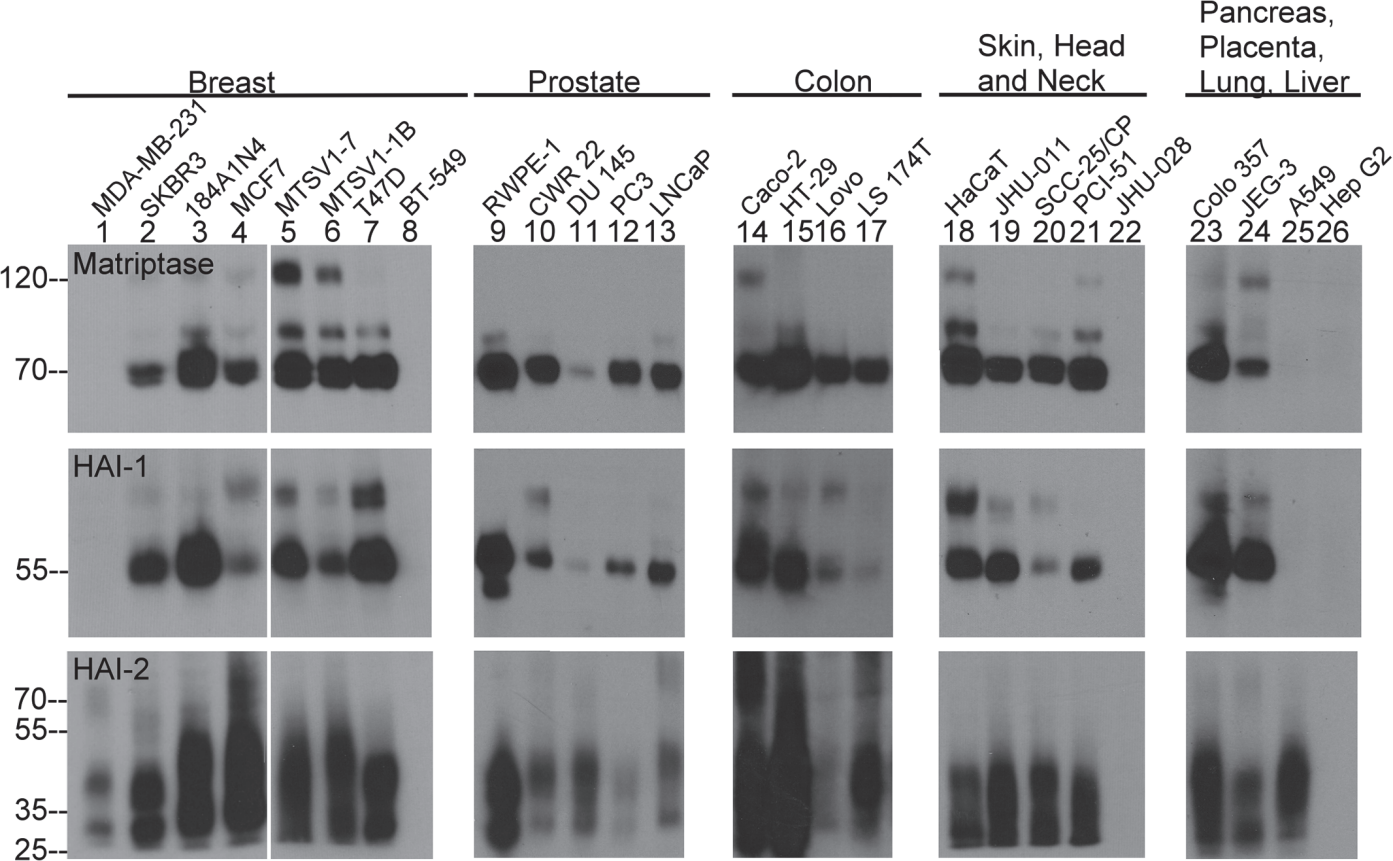
Co-expression of HAI-1 and HAI-1 with matriptase in human epithelial and carcinoma cells. Cell lysates were prepared from 26 human epithelial and carcinoma lines derived from the organ systems indicated. They were analyzed by immunoblot for the protein expression level of matriptase (upper panels), HAI-1 (middle panels), and HAI-2 (lower panels). Matriptase was detected as the 70-kDa zymogen, the 94-kDa full-length form, and a 120-kDa complex with HAI-1. HAI-1 was detected as the 55-kDa form and as an 85-kDa complex probably with bovine hepatocyte growth factor activator. HAI-2 was detected as a doublet band of between 25–45 kDa. Samples containing equal concentrations of cellular protein within each organ system were analyzed and then different amounts cellular protein in different organ systems were analyzed to provide the optimal levels shown in the immunoblot analysis presented.

The functional relationship between matriptase, HAI-1, and HAI-2 was first examined by determining the protein expression level in 26 human breast, prostate, colon, head and neck, skin, pancreas, placenta, lung, and liver epithelial or carcinoma cell lines (Fig. 1). Matriptase was detected principally in its 70-kDa mature zymogen form, though 94-kDa full-length matriptase and the 120-kDa matriptase-HAI-1 complex were also detected in some of these cell lines [10,26]. HAI-1 was detected mainly in its 55-kDa mature form, though other HAI-1 containing species were frequently detected which likely represent complexes of HAI-1with bovine HGFA or human prostasin [27]. HAI-2 was detected as a smeared band between roughly 25–45kDa which is probably due, in part, to its extensive N-glycosylation [1]. A 70-kDa HAI-2 species was also detected, which appeared to be more intense in some colon carcinoma cell lines (Fig. 1, lanes 14–17). Of the 26 cell lines examined, matriptase was detected in 21 lines in which HAI-1 and HAI-2 were also detected. Of the five lines that lacked detectable matriptase, all were also negative for HAI-1 expression whereas two expressed HAI-2. The Spearman Correlation Coefficients between matriptase and HAI-1 expression is 1 (ρ< 0.0001) and between matriptase and HAI-2 is 0.74015 (ρ< 0.0001). These correlation data are suggestive of a functional relationship between matriptase and HAI-1, and HAI-2.

### In solution, HAI-2 is a more potent matriptase inhibitor than HAI-1.

**Fig 2:**
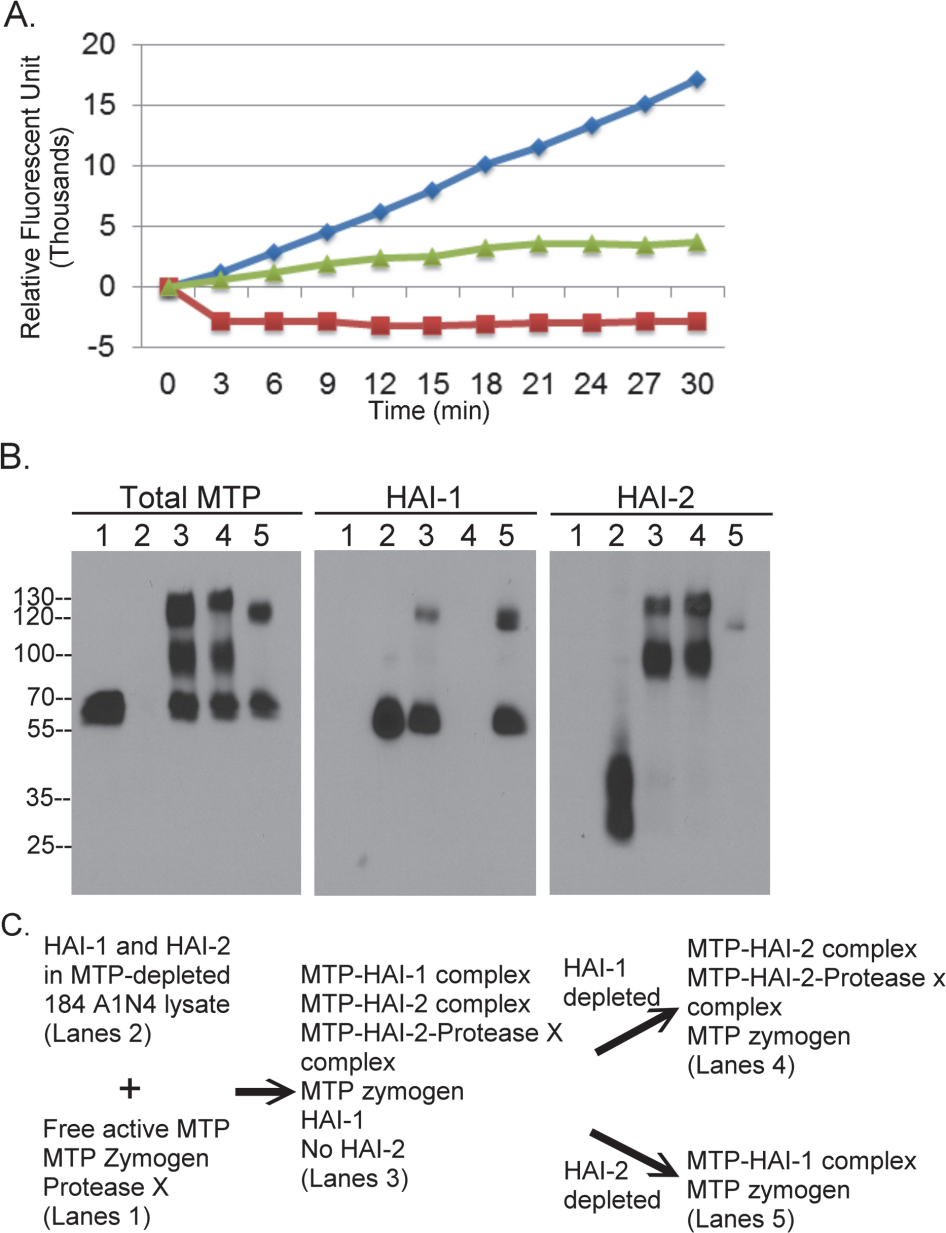
In Solution, HAI-2 is a better matriptase inhibitor than HAI-1. *A*. Active matriptase prepared from Ramos human B-cell lymphoma cells was mixed with 184 A1N4 human mammary epithelial cell lysate and incubated at 37°C for 10 min. Active matriptase preparation alone, cell lysate alone, and the mixture were analyzed for matriptase tryptic activity by cleavage of the fluorogenic substrate Boc-Gln-Ala-Arg-AMC. RFU stands for relative fluorescent units. *B*. The active matriptase preparation was mixed with matriptase-depleted 184 A1N4 cell lysates and at 37°C for 10 min to allow inhibition of matriptase by the HAIs. The mixture was divided and subjected to immunodepletion of HAI-1 species using the HAI-1 mAb M19 or HAI-2 species using the HAI-2 mAb DC16. The active matriptase preparation (lanes 1), the matriptase-depleted 184 A1N4 cell lysate (lanes 2), the mixture (lanes 3), the HAI-1 depleted mixture, and the HAI-2 depleted mixture were analyzed for matriptase species, HAI-1 species, and HAI-2 species by immunoblot using the matriptase mAb M24 (Total MTP), the HAI-1 mAb M19 (HAI-1) and the HAI-2 mAb DC16 (HAI-2), respectively.

Next, we compared the ability of cell-derived HAI-1 and HAI-2 to inhibit active matriptase. Free active matriptase along with matriptase zymogen was prepared from the material shed from Ramos human Burkitt lymphoma cells exposed to a pH 6.0 buffer, in order to induce matriptase activation. Active matriptase represents the vast majority of the tryptic activity in this shed fraction, which can be detected by cleavage of the synthetic fluorogenic substrate Boc-Gln-Ala-Arg-AMC (Fig. 2A, Blue Diamond), and which can be immunodepleted by a matriptase mAb, as demonstrated previously [28]. Both HAI-1 and HAI-2 were prepared from 184 A1N4 mammary epithelial cell lysates, from which the endogenous matriptase had been removed by immunodepletion using matriptase mAb 21–9 conjugated to Sepharose beads (Fig. 2B, lanes 2) and contained no tryptic activity capable of cleaving Boc-Gln-Ala-Arg-AMC (Fig. 2A Red Rectangle). Incubation of the 184 A1N4 preparation with the free active matriptase prepared from Ramos cells at 37°C for 10 minutes, resulted in a sample in which matriptase tryptic activity was significant inhibited (Fig. 2A, Green Triangle). Analysis of this sample by immunoblot assay demonstrated that the formation of matriptase complexes of around 100- and 120-kDa (Fig. 2B, Total MTP, lane 3) was associated with the suppression of tryptic activity. Using the HAI-1 mAb M19 and HAI-2 mAb DC16, we also showed that a HAI-1 complex of 120-kDa (Fig. 2B HAI-1, lane 3) and two HAI-2 complexes of 100- and 130-kDa had been formed (Fig. 2B HAI-2, lane 3). The 120-kDa matriptase-HAI-1 complex and the 130-kDa matriptase-HAI-2 complex appear as one large merged band in the immunoblot analysis using the matriptase mAb (Fig. 2B, Total MTP, lane 3). To further confirm the identity of these three HAI-matriptase complexes present in the sample (Fig. 2B, lanes 3) we either depleted the HAI-1 species from the sample with the HAI-1 mAb (Fig. 2B, HAI-1, lane 4) or depleted the HAI-2 species with the HAI-2 mAb (Fig. 2B, HAI-2, lane 5). Along with the depletion of HAI-1 species (Fig. 2B, HAI-1 lane 4), the lower portion of the 120-kDa matriptase complex was depleted (Fig. 2B, total MTP, lane 4). The specificity of the HAI-1 immunodepletion was confirmed by the continued presence of the 70-kDa matriptase zymogen and the two matriptase-HAI-2 complexes (Fig. 2B, total MTP, lane 4; HAI-2, lane 4). These analyses confirm that the lower portion of the 120-kDa matriptase containing complex is matriptase bound with HAI-1 and its presence indicates that matriptase is being inhibited by interacting with HAI-1. Similarly, concurrent with the immunodepletion of HAI-2 containing species (Fig. 2B, HAI-2, lane 5), the upper portion of the 120-kDa and the 100-kDa species detected with the matriptase antibody were removed (Fig. 2B, total MTP, lane 5), suggesting that these two matriptase complexes contain HAI-2. Remaining after HAI-2 immunodepletion was the 70-kDa matriptase zymogen, free HAI-1 and the 120-kDa matriptase-HAI-1 complex (Fig. 2B, lanes 5 under total MTP and HAI-1,) which again verifies the specificity of immunodepletion. These data suggest that the formation of the two matriptase-HAI-2 complexes contributes to the inhibition of active matriptase. These data also imply that HAI-2 may be a more potent inhibitor than HAI-1 in solution. We base this supposition on the relative ratio of the abundance of the matriptase-HAI-1 complex versus matriptase-HAI-2 complexes compared to the ratio of the uncomplexed HAI-1 versus HAI-2. The matriptase-HAI-1:matriptase-HAI-2 ratio was determined to be around 1:2.5 by densitometry and analysis using ImageJ of the bands representing the 120-kDa (Fig. 2B, MTP, lane 5), and the 100-, and 130-kDa (Fig. 2B, total MTP, lane 4) matriptase complexes followed by normalization of these values by the value for the 70-kDa matriptase zymogen bands (Fig. 2B, MTP, lanes 4 and 5). In addition we observed that there is very little free HAI-2, the majority being bound to active matriptase to form the two complexes (Fig. 2B, HAI-2 lane 3), whereas only 18% of the available HAI-1 was bound to matriptase in the 120-kDa complex (Fig. 2B, HAI-1, lane 3). Thus, in 184 A1N4 cells the amount of HAI-1 is about 2.2 (1/2.5÷0.18 = 2.2) times greater than HAI-2 prior to the incubation with active matriptase, which is in marked contrast to the matriptase-HAI-1:matriptase-HAI-2 ratio (1:2.5) after incubation with active matriptase. Collectively, these data suggest that both HAI-1 and HAI-2 are capable forming stable complexes with active matriptase in solution and that cellular HAI-2 appears to be a more effective matriptase inhibitor than cellular HAI-1. In addition, given the molecular masses of the proteins, the stoichiometry of the matriptase complexes appears to be 1:1 for the 100-kDa matriptase-HAI-2 complex and the 120-kDa matriptase-HAI-1complex. It seems likely that there must be a third protein in addition to matriptase in the 130-kDa HAI-2 complex. We summarize the experiment in Fig. 2C.

### Role of HAI-2 in matriptase inhibition in immortalized mammary epithelial cells versus breast cancer cells

Given the strong correlation of matriptase expression with HAI-2 (Fig. 1) and the greater potency for matriptase inhibition than cellular HAI-1 (Fig. 2), one might expect that HAI-2 is an important endogenous matriptase inhibitor. To examine this we studied the formation of matriptase-HAI-2 complexes following pH 6.0-induced matriptase activation by immunoblot using either a matriptase mAb or HAI-2 mAb. A variety of immortalized human epithelial cells and carcinoma cells were tested, including the 26 lines described in Fig. 1. We have found that in different types of epithelium and in different contexts HAI-2 appears to play various roles in matriptase inhibition. In this study, we focus on the differential role of HAI-2 in mammary epithelial cells compared to breast cancer cells.

**Fig 3:**
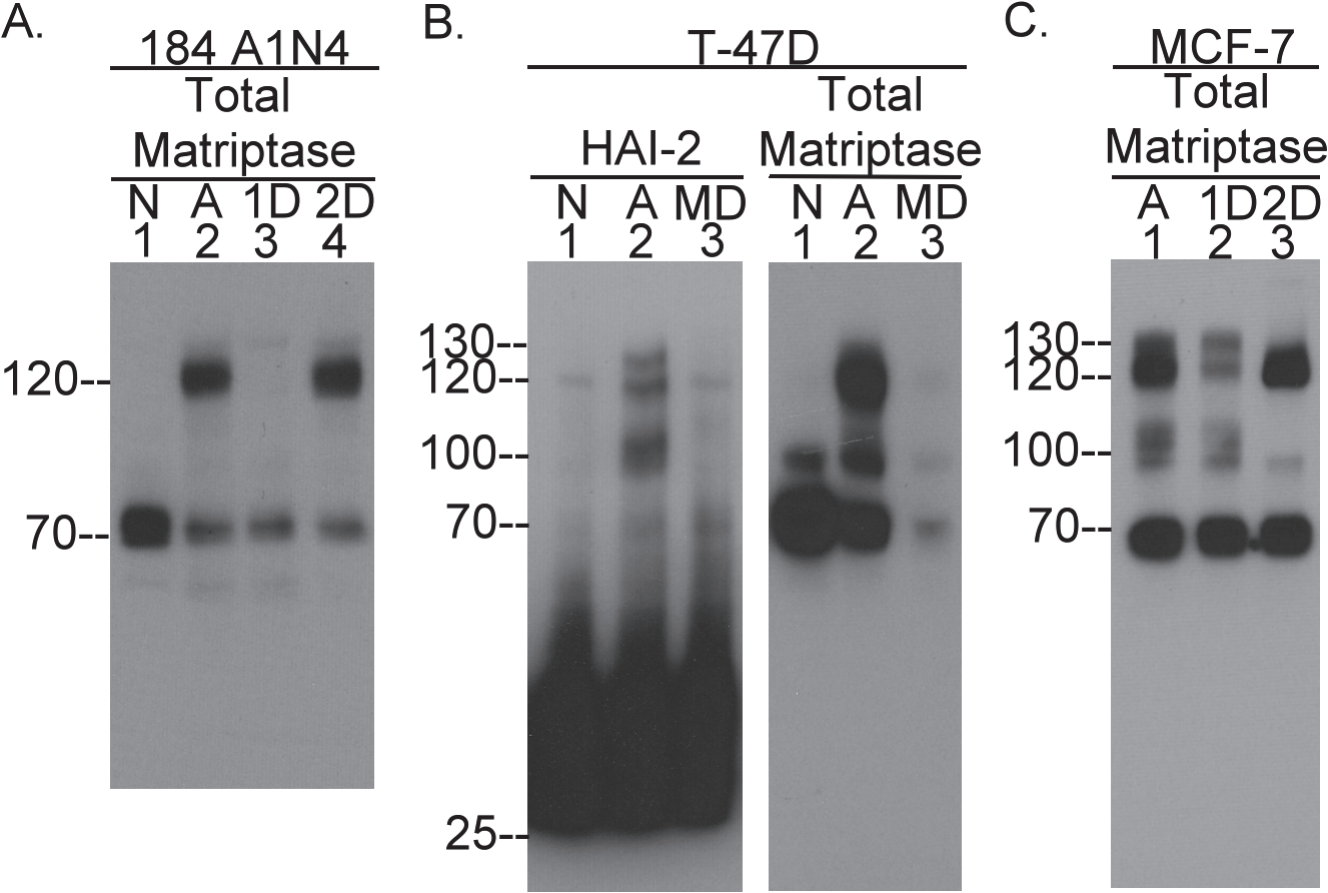
HAI-2 can inhibit matriptase in breast cancer cells but not mammary epithelial cells. *A*. 184 A1N4 cells were transiently exposed to a pH 6.0 buffer to induce matriptase zymogen activation. Cell lysates were prepared and subjected to immunodepletion using the HAI-1 mAb M19 or the HAI-2 mAb DC 16. The non-activation control lysate (lane 1) and the post-activation cell lysate (lane 2), the HAI-1-depleted lysate (lane 3) and the HAI-2-depleted lysate (lane 4) were analyzed for matriptase species by immunoblot. *B*. T-47D breast cancer cells were exposed to a pH 6.0 buffer to induce matriptase zymogen activation. Cell lysates were prepared and subjected to immunodepletion to remove matriptase. Lysate from the cells treated with PBS buffer as the non-activation control (lanes 1), lysate from the cells after induction of matriptase activation (lanes 2), and lysate after depletion of matriptase species (lanes 3) were analyzed by immunoblot for HAI-2 (left panel) and total matriptase (right panel). *C*. MCF7 breast cancer cells were exposed to the pH 6.0 buffer to induce matriptase zymogen activation (lane 1). Cell lysates were prepared and subjected to immunodepletion by using the HAI-1 mAb M19 (lane 2) or the HAI-2 mAb DC 16 (lane 3). These three samples were analyzed by immunblot for matriptase species.

Induction of matriptase zymogen activation in 184 A1N4 human mammary epithelial cells by transient exposure of the cells to a pH 6.0 buffer resulted in the conversion of the 70-kDa matriptase zymogen (Fig. 3A, lane 1) to a 120-kDa activated matriptase-HAI-1 complex but no 100-kDa matriptase-HAI-2 complex was detected (Fig. 3A, lane 2). Incubation of the lysate with HAI-1 mAb M19-Sepharose resulted in the complete immunodepletion of the 120-kDa matriptase complex from the lysate (Fig. 3A, lane 3). While there are some very weak matriptase bands at 100- and 130-kDa (Fig. 3A, lane 2), neither these weak signals or the 120-kDa matriptase complex were immunodepleted by incubation with HAI-2 mAb DC16-Sepharose (Fig. 3A, lane 4). These data suggest that in intact human mammary epithelial cells, HAI-1, but not HAI-2, is the predominant endogenous inhibitor of matriptase activity. This is in marked contrast to the situation in solution, in which context HAI-2 is a more potent inhibitor than HAI-1 (Fig. 2). The 120-kDa matriptase-HAI-1 complex was also the predominant product following the induction of matriptase zymogen activation in several non-turmorogenic human mammary epithelial cells used in our previous study [21], including the lines MCF-10A, MTSV 1–1B, and MTSV 1–7. No 100-kDa matriptase complex was detected in lysates prepared from these cells after the induction of matriptase zymogen activation [21]. Collectively, our previous and current results suggest that HAI-1, but not HAI-2, is the predominant inhibitor of matriptase in human mammary epithelial cells.

Several lines of evidence suggest that HAI-2 can play a role in the control of matriptase activity in human breast cancer cells. The detection of matriptase-HAI-2 complexes is, however, not as straightforward as the detection of matriptase-HAI-1 complexes. Matriptase-HAI-2 complexes are generally of low abundance and so the 130-kDa species is easily masked by the much more abundant 120-kDa matriptase-HAI-1 complex. The ratio of matriptase-HAI-2 complex to uncomplexed HAI-2 is also very low and as such matriptase-HAI-2 complexes are not easily detected by the HAI-2 mAb DC16. Nevertheless, in human T-47D human breast cancer cells, induction of matriptase activation by exposure to pH 6.0 buffer resulted in the formation of three HAI-2 complexes of 100-, 120-, and 130-kDa along with the formation of the 120-kDa matriptase-HAI-1 complex (Fig. 3B, comparing lanes 2 with lanes 1). The three HAI-2 complexes along with the 120-kDa matriptase-HAI-1 complex were all immunodepleted by matriptase mAb 21-9-Sepharose (Fig. 3B, lanes 3). The specificity of the immunodepletion was verified by the unaltered level of uncomplexed HAI-2 (Fig. 3B, HAI-2, lane 3). These data suggest that all of these HAI-1 and HAI-2 complexes contain matriptase. The formation of three matriptase-HAI-2 complexes and the matriptase-HAI-1 complex was also observed in MCF-7 breast cancer cells following induction of matriptase activation by a pH 6.0 buffer (Fig. 3C, lane 1). The three matriptase-HAI-2 complexes were readily detected after immunodepletion of the 120-kDa matriptase-HAI-1 complex using HAI-1 mAb M19-Sepharose (Fig. 3C, lane 2). Conversely, the 120-kDa matriptase-HAI-1 remained after the removal of the HAI-2 species by immunodepletion using HAI-2 mAb DC16-Sepharose (Fig. 3C, lane 3). These data suggest that HAI-2 acts in concert with HAI-1 to function as an inhibitor of matriptase in breast cancer cells.

### In mammary epithelial cells, matriptase zymogen activation occurs at cell-cell junctions whereas HAI-2 is intracellularly located.

**Fig 4:**
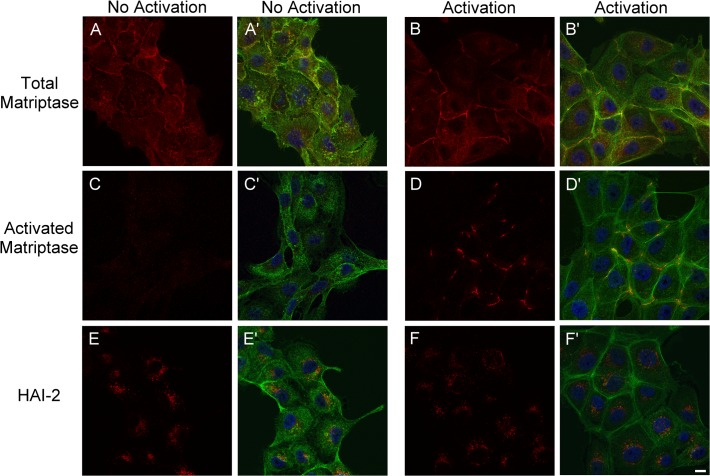
The different subcellular localizations of HAI-1 and HAI-2 result in the ability of HAI-1 but not HAI-2 to control matriptase activity in mammary epithelial cells. Human mammary epithelial cells 184 A1N4 were exposed to fresh culture medium for 30 min to induce matriptase zymogen activation. The non-activation control (*A, C*, and *E*, red) and the treated cells (*B, D*, and *F*, red) were subjected to indirect immunofluorescent staining using the total matriptase mAb M24 (*A* and *B*, red), the activated matriptase mAb M69 (*C* and *D*, red), and HAI-2 mAb DC16 (*E* and *F*). The cells were also counterstained with Alexa Fluor 488 labeled phalloidin for F-actin (green) and DAPI for nuclei (blue). Merged images are presented in *A’, B’,C’, D’, E’ and F’*. Scale bar: 10 μm.

To begin to explain why HAI-2 does not appear to play a significant role in the control of matriptase activity in mammary epithelial cells we wondered if cellular compartmentalization prevents HAI-2 from interacting with activated matriptase in these cells. The subcellular distribution of total and activated matriptase, and HAI-2 was determined by fluorescent immunocytochemistry in 184 A1N4 human mammary epithelial cells before and after the induction of matriptase zymogen activation by exposure to sphingosine 1-phosphate in serum, as described previously [24,25,29]. The cells were also counter-stained with the nuclear stain DAPI (blue) Alexa Fluor 488 labeled phalloidin (green) to visualize changes in cell shape and actin organization (Fig. 4A’–F’). Prior to the exposure to the fresh serum-containing culture medium, matriptase was detected primarily as a diffuse signal with some staining at the cell-cell contacts (Fig. 4A and A’), and HAI-2 was primarily detected in perinuclear granular/vesicle structures (Fig. 4E, and E’). There was no detectable staining for activated matriptase (Fig. 4C and C’). Thirty minutes after the medium was changed (serum exposure), in concert with the assembly cortical actin and formation of adherens junctions (Fig. 4, B’, D’, and F’, Green) [29], a significant proportion of the total matriptase staining translocated to the newly formed cell-cell junctions and accumulated in the middle of two cortical actin bands between two individual cells (Fig. 4B and B’). Activated matriptase induced by the serum exposure was detected between the bands of cortical actin at cell-cell junctions (Fig. 4D and D’), in the same area where the total matriptase accumulated the most. The co-translocalization of HAI-1 with matriptase to cell-cell junctions when matriptase zymogen activation is induced by sphinogosine 1-phosphate (or serum exposure) has been shown in our previous study [11]. In contrast, however, the HAI-2 remained in the granules/vesicles observed in the untreated cells after serum treatment (Fig. 4F and F’). These data reveal that HAI-2 is prevented from interacting with and inhibiting activated matriptase in mammary epithelial cells.

### HAI-2 accumulates on the surface of breast cancer cells, where it gains access to active matriptase

**Fig 5:**
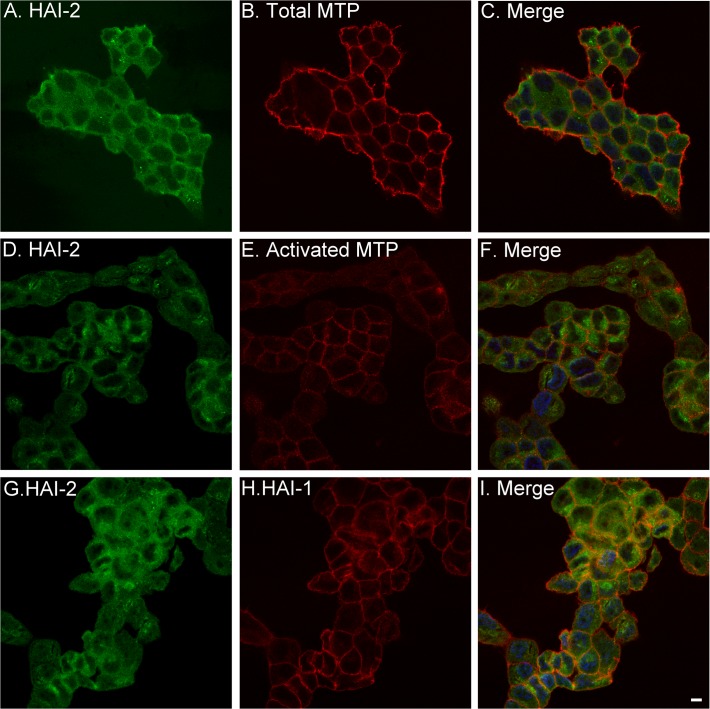
Misrouting of HAI-2 to the surface of breast cancer cells allows the inhibitor to access matriptase. The coloclaization of HAI-2 with matriptase (*A-C*), activated matriptase (*D-F*), and HAI-1 (*G-I*) in T-47D human breast cancer cells was analyzed by immunofluorescent double staining with (*D*-*I*) and without (*A*-*C*) permeabilization of the cells. The cells were either untreated so that matriptase was in the zymogen form (*A, B*, and *C*) or induced to activate matriptase by exposure to a pH 6.0 buffer (*D*-*I*). The nuclei were counter-stained with DAPI (blue). Scale bar: 10 μm.

The rapid formation of matriptase-HAI-2 complexes in breast cancer cells suggests that the subcellular distribution of HAI-2 and/or matriptase is different than that found in mammary epithelial cells, such that matriptase and HAI-2 reside within the cell with sufficient proximity to allow the rapid inhibition to occur. Significant HAI-2 signal was detected on the surface of the breast cancer cells when non-permeabilizing conditions are used for immunofluorescent staining (Fig. 5A). Prior to the induction of matriptase zymogen activation, matriptase was stained primarily on the periphery of the breast cancer cells under the same conditions (Fig. 5B). The coincidence of HAI-2 with matriptase on the surface of breast cancer cells (Fig. 5C) is consistent with HAI-2 having access to matriptase. We next induced matriptase activation by transiently exposing the breast cancer cells to a pH 6.0 buffer to assess the coincidence of HAI-2 and activated matriptase (Fig. 5D, E and F) by immunofluorescent staining under permeabilizing condition, which allowed the detection of intracellular HAI-2. Much of the activated matriptase was focused on the cell-cell junctions, where the matriptase zymogen was found prior to the induction of activation (Fig. 5E), suggesting that breast cancer cells activate matriptase at the cell-cell junction. The staining for HAI-2 was in general brighter in permeabilzed cells (Fig. 5D and G), and though more diffuse, HAI-2 staining was readily observed at the cell-cell junctions and coincided with the activated matriptase (Fig. 5D, E and F). The diffuse staining pattern indicates that HAI-2 could be broadly distributed within breast cancer cells. In contrast, HAI-1 staining was much more focused at the cell periphery (Fig. 5H) with the staining pattern almost identical to that of total matriptase (Fig. 5B) and activated matriptase (Fig. 5E). The staining patterns indicate that HAI-1 probably has better access to active matriptase than HAI-2, which is supported by the higher levels of matriptase-HAI-1 complex than matriptase-HAI-2 complexes detected by immunoblot analysis (Fig. 3). It is worthwhile noting that HAI-2 was also detected in intracellular granules in a few breast cancer cells but at a different plain than the cell surface. The granule staining was seen only in some cells (Fig. 5F and I).

## Discussion

The signal peptides and hydrophobic regions within their amino acid sequences indicate that HAI-2, like HAI-1, undergo synthesis and post-translational modification through the secretory pathway and become anchored in the cell membrane. This similarity combined with the practically identical amino acid sequence of VGRCR(A/G)S at the reactive center of the Kunitz domain 1, suggests that HAI-2 should be able to inhibit matriptase in a very similar manner to HAI-1. Our current study, however, suggests that the subcellular targeting of HAI-2 is a more important determinant of whether HAI-2 can inhibit matriptase in context of the live cell. The rapid inhibition of matriptase by HAI-1 only allows matriptase to be inhibited by other proteases inhibitors when these protease inhibitors are also co-localized with matriptase. This seems to be the case in breast cancer cells in which HAI-1 and HAI-2 work in concert to inhibit matriptase. The greater potency of HAI-2 against matriptase compared to HAI-1 in solution also appears to contribute to its ability to inhibit matriptase in the face of high levels of HAI-1. The inhibition of matriptase by HAI-2, however, appears to be a cancer-specific event and does not occur in mammary epithelial cells, in which HAI-2 exhibits intracellular localization in as yet un-identified granular structures rather than at cell-cell junctions where the majority of the activated matriptase accumulates.

The presence of activated matriptase predominantly on the cell surface of breast cancer cells suggests that matriptase zymogen activation and the inhibition of active matriptase by HAI-2 and HAI-1 takes place on the cell surface. Alternatively, matriptase may be activated and then inactivated by binding with the HAIs inside the cells in the secretory pathway, and the newly formed matriptase-HAI complexes then rapidly translocate to the cell-cell junctions. In either case, free active matriptase only has very short time to act on its downstream substrates prior to HAI-mediated inhibition. Although a proportion of the active matriptase is shed into the extracellular milieu [28], where it could activate pro-uPA, pro-HGF, PDGF-D and other extracellular substrates [30,31], a significant amount of the active matriptase must act on its substrates in the face of the overwhelming concentrations of the HAIs. Previously we have shown that in human keratinocytes the short-lived, cell-associated free active matriptase is able to activate prostasin prior to its inhibition by HAI-1 [27]. The generation of the 120- and 130-kDa matriptase-HAI-2 complexes, species larger than can be explained by the presence of matriptase and HAI-2 alone, suggests that some other proteases has been activated along with the activation of matriptase, and is bound to the other HAI-2 Kunitz domain. It is tempting to hypothesize that this protease(s) is a matriptase substrate in breast cancer cells. A 130-kDa matriptase-HAI-2 complex was also formed in solution when active matriptase prepared from lymphoma cells was incubated with the HAIs in solution. This 130-kDa matriptase-HAI-2 complex may be similar to or identical to the complex formed in breast cancer cells, suggesting that the activation of other proteases along with the induction of matriptase zymogen activation may also occur in lymphoma cells. Purification and protein identification of the components of these matriptase-HAI-2 complexes should provide the insights to the function of the shorted-lived cell-associated active matriptase and the significance of the activation of these proteases in cancer. Specifically, breast cancer cells have been known to constitutively activate matriptase [32]. By considering that breast cancer cells lose epithelial polarity, the increased matriptase activity might gain access to some new substrates, likely including those proteases in the complexes with HAI-2 and matriptase. The increased proteolysis could, however, have negative effect on breast cancer cells. The increased levels of cell surface HAI-2 might provide a means for breast cancer cells to control the increased pericellular proteolysis.

In addition to matriptase, HAI-2 may also inhibit other cellular proteases as suggested by the detection of HAI-2 species with apparent molecular masses greater than HAI-2. These high molecular weight HAI-2 species likely represent HAI-2 complexed with serine proteases other than matriptase. Their detection is generally not easy in most epithelial and carcinoma cells due to their low abundance and/or the ratio between HAI-2 and the HAI-2 complexes. The level of these HAI-2 complexes appears to be much higher in some colon carcinoma cells than in cancer cells from other organs. Furthermore, the formation of these complexes is apparently constitutive rather than induced by acid exposure as seen with the formation of complexes with matriptase. The constitutive formation of these HAI-2 complexes suggests that colon carcinoma cells might constitutively activate some serine proteases, which may represent the genuine and predominant target proteases for HAI-2 in these cells. These proteases may, therefore, be particularly active in colon carcinoma cells compared to epithelial and carcinoma cells in other organ systems.

In summary, although HAI-2 significantly resembles HAI-1 with respect to overall protein domain structure and protease inhibitory specificity, the role of HAI-2 in the control of matriptase is not as ubiquitous and straightforward as is the case for HAI-1. Whether HAI-2 is or can become co-localized with matriptase appears to be the major determinant for the importance of HAI-2 in the control of matriptase activity. Although the mechanism by which HAI-2 subcellular targeting is regulated remains largely unexplored, the physiological mechanism employed by mammary epithelial cells appears to be dysregulated in breast cancer cells. As a consequence, HAI-2 participates in the control of matriptase activity in breast cancer cells but not in mammary epithelial cells. Our study indicates that the functional relationship between protease and protease inhibitor is not linear and that cellular compartmentalization can prevent one protease inhibitor from having access to a protease while facilitating the access of another protease inhibitor.

## References

[1] Kawaguchi T, Qin L, Shimomura T, Kondo J, Matsumoto K, Denda K, et al Purification and cloning of hepatocyte growth factor activator inhibitor type 2, a Kunitz-type serine protease inhibitor. J Biol Chem. 1997;272:27558–27564. 9346890 10.1074/jbc.272.44.27558

[2] Shimomura T, Denda K, Kitamura A, Kawaguchi T, Kito M, Kondo J, et al Hepatocyte growth factor activator inhibitor, a novel Kunitz-type serine protease inhibitor. J Biol Chem. 1997;272:6370–6376. 9045658 10.1074/jbc.272.10.6370

[3] Kataoka H, Suganuma T, Shimomura T, Itoh H, Kitamura N, Nabeshima K, et al Distribution of hepatocyte growth factor activator inhibitor type 1 (HAI-1) in human tissues. Cellular surface localization of HAI-1 in simple columnar epithelium and its modulated expression in injured and regenerative tissues. J Histochem Cytochem. 1999;47:673–682. 10219059 10.1177/002215549904700509

[4] Szabo R, Hobson JP, List K, Molinolo A, Lin CY, Bugge TH. Potent inhibition and global co-localization implicate the transmembrane kunitz-type serine protease inhibitor hai-2 in the regulation of epithelial matriptase activity. J Biol Chem. 2008;283: 29495–504. 10.1074/jbc.M801970200 18713750 PMC2570866

[5] Miyazawa K, Shimomura T, Kitamura A, Kondo J, Morimoto Y, Kitamura N. Molecular cloning and sequence analysis of the cDNA for a human serine protease reponsible for activation of hepatocyte growth factor. Structural similarity of the protease precursor to blood coagulation factor XII. J Biol Chem. 1993;268:10024–10028. 7683665

[6] Lin CY, Anders J, Johnson M, Dickson RB Purification and characterization of a complex containing matriptase and a Kunitz-type serine protease inhibitor from human milk. J Biol Chem. 1999;274:18237–18242. 10373425 10.1074/jbc.274.26.18237

[7] Wang JK, Lee MS, Tseng IC, Chou FP, Chen YW, Fulton A, et al Polarized epithelial cells secrete matriptase as a consequence of zymogen activation and HAI-1-mediated inhibition. Am J Physiol Cell Physiol. 2009;297:C459–C470. 10.1152/ajpcell.00201.2009 19535514 PMC2724094

[8] Kirchhofer D, Peek M, Li W, Stamos J, Eigenbrot C, Kadkhodayan S, et al Tissue expression, protease specificity, and Kunitz domain functions of hepatocyte growth factor activator inhibitor-1B (HAI-1B), a new splice variant of HAI-1. J Biol Chem. 2003;278:36341–36349. 12815039 10.1074/jbc.M304643200

[9] Oberst MD, Singh B, Ossandon M, Dickson RB, Johnson MD, Lin CY. Characterization of matriptase expression in normal human tissues. J Histochem Cytochem. 2003;51: 1017–1025. 12871983 10.1177/002215540305100805

[10] Oberst MD, Williams CA, Dickson RB, Johnson MD, Lin CY. The activation of matriptase requires its noncatalytic domains, serine protease domain, and its cognate inhibitor. J Biol Chem. 2003;278:26773–26779. 12738778 10.1074/jbc.M304282200

[11] Oberst MD, Chen LY, Kiyomiya KI, Williams CA, Lee MS, Johnson MD, et al Hepatocyte growth factor activator inhibitor 1 (HAI-1) regulates activation and expression of matriptase, a membrane-bound serine protease. Am J Physiol Cell Physiol. 2005;289:C462–C470. 15800053 10.1152/ajpcell.00076.2005

[12] Szabo R, Hobson JP, Christoph K, Kosa P, List K, Bugge TH. Regulation of cell surface protease matriptase by HAI2 is essential for placental development, neural tube closure and embryonic survival in mice. Development. 2009;136:2653–2663. 10.1242/dev.038430 19592578 PMC2709071

[13] Xu H, Xu Z, Tseng IC, Chou FP, Chen YW, Wang JK, et al Mechanisms for the control of matriptase activity in the absence of sufficient HAI-1. Am J Physiol Cell Physiol. 2012;302:C453–C462. 10.1152/ajpcell.00344.2011 22031598 PMC3328841

[14] Heinz-Erian P, Muller T, Krabichler B, Schranz M, Becker C, Ruschendorf F, et al Mutations in SPINT2 cause a syndromic form of congenital sodium diarrhea. Am J Hum Genet. 2009;84:188–196. 10.1016/j.ajhg.2009.01.004 19185281 PMC2668003

[15] Itoh H, Kataoka H, Hamasuna R, Kitamura N, Koono M. Hepatocyte growth factor activator inhibitor type 2 lacking the first Kunitz-type serine proteinase inhibitor domain is a predominant product in mouse but not in human. Biochem Biophys Res Commun. 1999;255:740–748. 10049781 10.1006/bbrc.1999.0268

[16] Stampfer MR, Bartley JC. Induction of transformation and continuous cell lines from normal human mammary epithelial cells after exposure to benzo[a]pyrene. Proc Natl Acad Sci U S A. 1985;82:2394–2398. 3857588 10.1073/pnas.82.8.2394 PMC397564

[17] Bartek J, Bartkova J, Kyprianou N, Lalani EN, Staskova Z, Shearer M, et al Efficient immortalization of luminal epithelial cells from human mammary gland by introduction of simian virus 40 large tumor antigen with a recombinant retrovirus. Proc Natl Acad Sci U S A. 1991;88:3520–3524. 1708884 10.1073/pnas.88.9.3520 PMC51483

[18] Teicher BA, Holden SA, Kelley MJ, Shea TC, Cucchi CA, Rosowsky A, et al Characterization of a human squamous carcinoma cell line resistant to cis-diamminedichloroplatinum (II). Cancer Res. 1987;47:388–393. 3539321

[19] Yasumura S, Hirabayashi H, Schwartz DR, Toso JF, Johnson JT, Herberman RB, et al Human cytotoxic T-cell lines with restricted specificity for squamous cell carcinoma of the head and neck. Cancer Res. 1993;53:1461–1468. 8443824

[20] Heo DS, Snyderman C, Gollin SM, Pan S, Walker E, Deka R, et al Biology, cytogenetics, and sensitivity to immunological effector cells of new head and neck squamous cell carcinoma lines. Cancer Res. 1989;49:5167–5175. 2766286

[21] Tseng IC, Xu H, Chou FP, Li G, Vazzano AP, Kao JP, et al Matriptase activation, an early cellular response to acidosis. J Biol Chem. 2010;285:3261–3270. 10.1074/jbc.M109.055640 19940125 PMC2823413

[22] Xu Z, Chen Y, Butta A, Wilder P, Weber D, Yu W, et al Targeting zymogen activation to control the matriptase-prostasin proteolytic cascade. J Med Chem. 2011;54:7567:7578. 10.1021/jm200920s 21966950 PMC3214968

[23] Lee MS, Kiyomiya K, Benaud C, Dickson RB, Lin CY. Simultaneous activation and HAI-1-mediated inhibition of matriptase induced at activation foci in immortal human mammary epithelial cells. Am J Physiol Cell Physiol. 2005;288:C932–C941. 15590895 10.1152/ajpcell.00497.2004

[24] Benaud C, Dickson RB, Lin CY. Regulation of the activity of matriptase on epithelial cell surfaces by a blood-derived factor. Eur J Biochem. 2001;268:1439–1447. 11231297 10.1046/j.1432-1327.2001.02016.x

[25] Benaud C, Oberst M, Hobson JP, Spiegel S, Dickson RB, Lin CY. Sphingosine 1-phosphate, present in serum-derived lipoproteins, activates matriptase. J Biol Chem. 2002;277:10539–10546. 11792696 10.1074/jbc.M109064200

[26] Cho EG, Kim MG, Kim C, Kim SR, Seong IS, Chung C, et al N-terminal processing is essential for release of epithin, a mouse type II membrane serine protease. J Biol Chem. 2001;276:44581–44589. 11567025 10.1074/jbc.M107059200

[27] Chen YW, Wang JK, Chou FP, Chen CY, Rorke EA, Chen LM, et al Regulation of the matriptase-prostasin cell surface proteolytic cascade by hepatocyte growth factor activator inhibitor-1 (HAI-1) during epidermal differentiation. J Biol Chem. 2010;285:31755–31762. 10.1074/jbc.M110.150367 20696767 PMC2951247

[28] Chu LL, Xu Y, Yang JR, Hu YA, Chang HH, Lai HY, et al Human Cancer Cells Retain Modest Levels of Enzymatically Active Matriptase Only in Extracellular Milieu following Induction of Zymogen Activation. PLoS One. 2014;9:e92244 10.1371/journal.pone.0092244 24663123 PMC3963879

[29] Hung RJ, Hsu I, Dreiling JL, Lee MJ, Williams CA, Oberst MD, et al Assembly of adherens junctions is required for sphingosine 1-phosphate-induced matriptase accumulation and activation at mammary epithelial cell-cell contacts. Am J Physiol Cell Physiol. 2004;286:C1159–C1169. 15075215 10.1152/ajpcell.00400.2003

[30] Chou FP, Chen YW, Zhao XF, Xu-Monette ZY, Gartenhaus RB, et al Imbalanced matriptase pericellular proteolysis contributes to the pathogenesis of malignant B-cell lymphomas. Am J Pathol. 2013;183:1306–1317. 10.1016/j.ajpath.2013.06.024 24070417 PMC3791685

[31] Ustach CV, Huang W, Conley-LaComb MK, Lin CY, Che M, Abrams J, et al A novel signaling axis of matriptase/PDGF-D/ss-PDGFR in human prostate cancer. Cancer Res. 2010;70:9631–9640. 10.1158/0008-5472.CAN-10-0511 21098708 PMC3058856

[32] Benaud CM, Oberst M, Dickson RB, Lin CY. Deregulated activation of matriptase in breast cancer cells. Clin Exp Metastasis. 2002;19:639–649. 12498394 10.1023/a:1020985632550

